# Future destinations: how people cured of hepatitis C using direct acting antiviral drugs progress in a new HCV-free world. A thematic analysis

**DOI:** 10.1186/s12954-024-01142-3

**Published:** 2025-01-16

**Authors:** Sarah R. Donaldson, Andrew Radley, John F. Dillon

**Affiliations:** 1 School of Medicine, University of Dundee, Dundee, DD1 9SY UK; 2 NHS Tayside, Dundee, DD1 9SY UK

**Keywords:** Hepatitis C, Direct-Acting Antivirals, Recovery, Identity

## Abstract

**Background:**

The introduction of Direct-Acting Antivirals (DAAs) transformed Hepatitis C (HCV) treatment, despite this uptake of DAAs remains lower than required to meet the WHO Sustainable Development Goal (3.3). Treatment with interferon was suggested to be able to deliver important outcomes for people who use drugs in addition to a viral cure, such as social redemption, and shift from a stigmatised identity. There is a lack of understanding if DAAs can deliver these transformative outcomes.

**Methods:**

This recurrent cross-sectional study combines qualitative semi-structured interviews and demographic data of 15 participants receiving DAAs in Tayside, Scotland. A thematic analysis explored the non-clinical outcomes of DAA treatment viewed through the lens of the Social Identity Model of Recovery (SIMOR) to build understanding of the influence DAAs have in a recovery journey from drug use.

**Results:**

Three key themes emerged: identity, relationships and social networks; building recovery capital; and reflecting on re-infection and the shift to DAAs. Concern about the transmission of HCV resulted in self-imposed isolation which weakened support structures. Cure provides a mechanism to strengthen family bonds, however social networks in the wider community remain limited. Participants gained opportunities to undertake activities that build health and wellbeing providing a shift in identity, future plans and aspirations. Social isolation remained for some, revealing unmet need in post-cure support.

**Conclusion:**

DAAs may support recovery journeys through the SIMOR, individuals reduced the number of active users within their social network and reconnected with family members, building recovery capital. Individuals, however, remained socially isolated in the context of the wider community. HCV services should support links to community resources to deliver the social inclusion people desire.

**Supplementary Information:**

The online version contains supplementary material available at 10.1186/s12954-024-01142-3.

## Introduction

Hepatitis C is a bloodborne virus which is strongly linked to injecting drug use due to the risk of transmission through this activity [1, 2]. Around 30% of people who contract the hepatitis C virus (HCV) will spontaneously clear it, with the remaining 70% requiring treatment. If left untreated HCV can result in chronic liver disease, cirrhosis and cancer [2].

Advances in treatment of HCV from interferon-based therapies to direct acting antivirals (DAAs) have transformed the experience of those requiring treatment [2]. This is especially true for the key risk group for HCV, people with a history of injecting drug use [1]. Interferon-based therapies have a high treatment burden, requiring long treatment periods with significant side effects and moderate cure rates (50–70%) [3]. These factors contributed to multiple structural, patient and provider barriers to accessing treatment, placing it out of reach for many people who inject drugs (PWID). However, for PWID who are able to negotiate these barriers literature describe the transformative experience of HCV treatment by supporting people towards a “normal life”, leaving drug use behind them [4–8]. This shift is attributed to the heavy treatment burden of interferon-based therapies and people feeling they had earned their cure [6, 9–11].

The introduction of direct-acting antiviral regimes provides a reliable opportunity for a cure (> 95%) in a relatively quick, oral format, with few side effects [12]. The availability of DAAs provides the backbone for the World Health Organization’s target to eliminate HCV as a public health concern by 2030 and countries are urged to embrace these medicines to achieve this ambition [13].

Despite advances in treatment the number of people engaging with HCV testing and treatment remains below that needed to achieve elimination in many countries [14–16]. Many countries continue to impose restrictive barriers to accessing DAAs, including on reimbursement for treatment and prescribing criteria. Additionally, a small number of nations still enforce restrictions relating to reinfection and drug use, further hindering access [17]. Stigma, competing priorities and the legacy of interferon continue to influence the perspectives of PWID in coming forward and accepting treatment [18]. The additional perspective from a social viewpoint, of how DAAs may support life plans may provide further compelling reasons to engage in HCV treatment and elimination [5].

DAA treatment provides a reliable cure, for a large majority of people, with a relatively small treatment burden, but they may not provide a sense of people “earning” their cure. As such they may have a lesser impact on rehabilitation and recovery from drug use with an increasing focus on the pill to eliminate the virus rather than deliver the outcomes PWID may desire [19]. A number of authors document the hopes and expectations of HCV treatment by PWID to include social redemption and an untainted identity away from the stigma surrounding drug use and HCV; a recovery journey [20, 21].

Recovery from drug use has different meanings to different people [22, 23]. In recent years, the view of recovery has shifted from viewing abstinence as achieving recovery to embracing recovery as a process aimed at building a better life, which may still involve some drug use [22]. This journey is marked by progress in areas such as quality of life, wellbeing, building and maintaining relationships and social inclusion [24–26].

Recovery capital refers to the resources that an individual can access to initiate and sustain their recovery journey [27]. The Social Identity Model of Recovery (SIMOR) provides a framework suggesting that recovery is supported by a shift in self-identity, which then shapes the social network of the individual to one where drug use is not the social norm [28–31]. These new or reclaimed social networks may provide greater support for recovery with a focus on tangible assets such as relationships, work, and community involvement, which build recovery capital [32]. The relationship between self-identity and social identity reinforces each other. Our previous work identified the SIMOR as a mechanism through which DAAs may be able to deliver outcomes beyond a viral cure that PWID are suggested to desire [33, 34].

However, other authors caution that DAAs may fail to deliver on the “hype” and “promises” of a “better life” for some undergoing treatment [11, 20, 35]. Harris [11] highlights the importance of managing the expectations of those undergoing DAA treatment. A care model which is mainly focused on a medical cure may not provide sufficient support to reverse entrenched social views and experiences [35–37]. An increased focus on “what lies beyond a cure?” is encouraged for HCV care services to evolve and deliver patient hopes and expectations from treatment [20, 35, 36].

In this study we look beyond a medical cure of HCV to build an understanding of the views, meaning and value PWID place on DAA treatment. We utilise the SIMOR as a lens to look for additional insights for how HCV care can grow to deliver recovery outcomes in the post-cure pillar of the HCV treatment cascade [38, 39].

## Method

This is a recurrent cross-sectional study that integrates qualitative data to understand people’s views, meanings and values and demographic data to support the themes identified and enhance the validity of the analysis by providing context and characteristics.

We capture and describe the non-clinical outcomes of people treated for HCV with DAAs in NHS Tayside, a health board region in Scotland which is recognised as innovative in their approach to treat HCV, embracing treatment as prevention as the way forward to eliminate HCV [40]. The area proactively tests and treats people for HCV through low threshold community pathways and this has been key to breaking down treatment barriers [41, 42]. NHS Tayside announced it had reached its elimination target July 2020 and the participants in this study were recruited during this time [43, 44].

Attempts were made to follow up with participants for 2 years post treatment with DAAs at three time points (baseline, 1 year and 2 years +/- 6months) to investigate if views, meanings and values shift and evolve over time after HCV cure.

The estimated sample size for this study was based on theoretical and practical considerations. A review of qualitative studies within this field indicated a target sample size in the region of 15–25 participants. The study recruited at the lower end of the target sample due to the reduced availability of the sample universe arising from restrictions during the Covid-19 pandemic and as a result of reaching HCV elimination within the region, with fewer people requiring treatment.

A convenience sampling strategy was used due to practicality and cost-effectiveness. Eligible participants who met the inclusion criteria were recruited through the standard pathway of care for HCV in NHS Tayside by the hepatitis C nursing team across three sites covering the Tayside region. Prior to, during, or just after treatment with DAAs potential participants were informed of the study, given participant information sheets and if interested, consent gained to pass contact details to the researcher. The researcher contacted the participant, answered any questions, and gained informed consent. Interviews were conducted face-to-face or over the telephone to comply with covid-19 restrictions in place at the time and/or participant preference. Supermarket vouchers were provided as recompense for participants’ time, set at £10 for interviews one & two and £20 for the final interview three.

### Inclusion criteria

- Adults with a history of problematic substance use with chronic HCV infection and planned/current/recently finished treatment with DAAs.
- Any ethnic origin who can speak English.
- Participants must be willing to talk about and reflect on the experience with the phenomenon under study.
- Participants must be willing to have semi-structured interviews audio recorded.

Participants who were pregnant were excluded from the study as it is difficult to differentiate the effect of pregnancy in relation to a recovery journey from treatment for HCV as the potential catalyst for change.

### Data collection & management

#### Qualitative data

Qualitative data was captured using semi-structure interviews and researcher field notes. Researcher field notes were recorded to provide context and capture observations which may have influenced the participants’ response. The semi-structured interviews aimed to gather participants’ views of being treated with DAAs and the meaning and value they placed on receiving this treatment and how this may have influenced their recovery journey (Supplementary material Table 1). The same topic guide was used for follow-up interviews as a tool to reflect on how views may shift and evolve over time. The interviews were audio recorded and transcribed verbatim.

#### Demographic data

It is important to consider the context, characteristics and experiences of the people participating in the research to add meaning and understanding to the thematic analysis. To do this information was gathered to describe individuals social and personal circumstances using the following validated tools. The *World Health Organization Alcohol*,* Smoking and Substance Involvement Screening Test (WHO ASSIST 3.0)* was used to provide a measure of current drug use [45]. The *Short Warwick-Edinburgh Mental Wellbeing Scale (SWEMWBS)* provided a measure of wellbeing [46]. The *Social Identity Map- Ascertaining identity resources (SIM - AIRing)* was used to describe the participant’s social network [47]. Participant demographic information also captured age, gender, ethnic background, financial inclusion, household income, employment data, and information relating to current and/or previous treatment for problematic substance use, and prior hepatitis C infection.

### Ethical considerations & data management

Participants were anonymised using a study identification number which was used on all study documents and recordings to maintain confidentiality.

The data management system used for demographic data collection was a password protected Excel spreadsheet held on NHS Tayside secure servers. Qualitative data was managed using NVivo 12.

### Theoretical stance & data analysis

This paper adopts a critical realist perspective. All data was collected before conducting the thematic analysis. Data analysis followed Braun and Clarke’s [48] six phase approach to thematic analysis and was undertaken by two members of the research team (AR and SRD), if a consensus could not be reached a further research team member was available to consult (JFD). A descriptive thematic analysis of interview stage one established broad codes aligned with the semi-structured interview questions. These initial codes were then refined into themes, and then viewed within the context and characteristics captured by the demographic data, guided by the method set out in Richie & Spencer [49]. A conceptual analysis was then undertaken using the lens of SIMOR to deepen the understanding of stage one themes. Data from follow up interviews (stages two and three) were then coded by both researchers (AR and SRD) and compared to the themes identified from stage one, identifying similarities ad emerging patterns. The purpose of the follow up interviews was to explore if time after HCV cure shifts the value and meaning placed on DAA treatment and how this influences recovery journeys. The Standards for Reporting Qualitative Research (SPQR) 21 item tool was used to guide the reporting of this research to improve transparency through clear standards for qualitative research [50] (Supplementary material Table 2).

## Results

Between 4th November 2020 and 31st May 2023, we conducted 20 qualitative interviews with 15 participants who meet the inclusion criteria set out in the study protocol (two participants who consented to the study were withdrawn as one only partially completed the interview one data due to covid-19 restrictions and the second was deemed to be under the influence at the interview). 15 participants completed the first interview stage, four participants completed interview stage two, and only one participant then went on to complete interview stage three. Researchers attempted to reach participants for follow up interviews using the telephone number provided by the participant at interview stage one. Contact was attempted six times before the participant was recorded as lost to follow-up. The length of interviews ranged from 18 min to 30 min.

### Demographic data

Table 1 below describes the context and characteristics of the participants in this study. At the first interview the mean age of participants was 41.9 years (± 7.07), the majority of participants were male (*n* = 11) and most stated they lived alone (*n* = 12), with three reporting to live with a partner (rather than family members). All participants were unemployed at the first interview, and more than half of our participants were receiving DAA treatment due to re-infection (*n* = 8, 53%).

**Table 1:** Demographic data describing the characteristics and context of participants

Participant number	Interview number	Age at interview (years old)	Gender	Financial inclusion question answer*	WHO ASSIST 3.0 Q2 score	SWEMWBS (Score out of 35)	SIM-AIRing Categorising social network: Red- active user Blue – in recovery Green – non-user	Due to re-infection?
**1**	1 (face-to-face)	41	Female	2	18	Not complete	Total 7, Red 2, Blue 1, Green 4	Yes
	2 – withdraw	-		-	-	-	-	
	3 – withdraw	-		-	-	-	-	
**2**	1 (face-to-face)	45	Male	2	13	22	Total 3, Red 3, Blue 0, Green 0	No
	2 – lost to follow up	-		-	-	-	-	
	3- lost to follow up	-		-	-	-	-	
**3**	1 (face-to-face)	48	Male	1	15	31	Total 3, Red 3, Blue 0, Green 0	Yes
	2 (telephone)	49		3	14	33	Total 3, Red 1, Blue 1, Green 1	
	3 – lost to follow up	-		-	-	-	-	
***4***	*1 -withdrawn*	*-*	*-*	*-*	*-*	*-*	*-*	*-*
**5**	1 (face-to-face)	39	Male	3	22	24	Total 9, Red 6, Blue 0, Green 3	No
	2 – withdraw	-		-	-	-	-	
	3 – withdraw	-		-	-	-	-	
**6**	1 (face-to-face)	40	Female	4	17	14	Total 3, Red 0, Blue 2, Green 1	Yes
	2 – lost to follow up	-		-	-	-	-	
	3 – lost to follow up	-		-	-	-	-	
**7**	1 (telephone)	42	Male	2	12	23	Total 6, Red 0, Blue 0, Green 6	No
	2 – withdraw	-		-	-	-	-	
	3 – withdraw	-		-	-	-	-	
**8**	1 (telephone)	27	Female	4	25	20	Total 5, Red 3, Blue 2, Green 0	No
	2 – withdraw	-		-	-	-	-	
	3 – withdraw	-		-	-	-	-	
***9***	*1-withdrawn*	*-*	*-*	*-*	*-*	*-*	*-*	*-*
**10**	1 (telephone)	49	Male	5	22	21	Total 2, Red 2, Blue 0, Green 0	Yes
	2 - deceased	-		-	-	-	-	
	3 - deceased	-		-	-	-	-	
**11**	1 (telephone)	40	Male	5	15	14	Total 0, Red 0, Blue 0, Green 0	No
	2 – lost to follow up	-		-	-	-	-	
	3 – lost to follow up	-		-	-	-	-	
**12**	1 (telephone)	36	Male	2	12	30	Total 1, Red 0, Blue 0, Green 1	Yes
	2 (telephone)	37		2	18	31	Total 2, Red 0, Blue 0, Green 2	
	3 – lost to follow up	-						
**13**	1 (telephone)	50	Male	2	12	20	Total 1, Red 0, Blue 0, Green 1	Yes
	2 (telephone)	51		2	0	29	Total 0, Red 0, Blue 0, Green 0	
	3 – lost to follow up	-		-	-	-	-	
**14**	1 (telephone)	50	Male	3	29	15	Total 4, Red 1, Blue 2, Green 1	No
	2 (telephone)	51		3	21	19	Total 2, Red 0, Blue 1, Green 1	
	3 (telephone)	52		3	20	14	Total 1, Red 0, Blue 0, Green 1	
**15**	1 (telephone)	35	Female	4	14	19	Total 2, Red 0, Blue 2, Green 0	Yes
	2 – lost to follow up	-		-	-	-	-	
	3 – lost to follow up	-		-	-	-	-	
**16**	1 (telephone)	52	Male	2	8	31	Total 1, Red 0, Blue 1, Green 0	No
	2 – lost to follow up	-		-	-	-	-	
	3 – lost to follow up	-		-	-	-	-	
**17**	1 (telephone)	35	Male	3	26	28	Total 1, Red 0, Blue 1, Green 0	Yes
	2 – lost to follow up	-		-	-	-	-	
	3 – lost to follow up	-		-	-	-	-	

Participants 4 and 9 were withdrawn due to exclusion criteria

*Financial comfort question “How well would you say you yourself are managing financially these days?”

Answer (1) Living comfortably, (2) Doing alright, (3) Just about getting by, (4) Finding it quite difficult, (5) Finding it very difficult

### Thematic analysis

Our thematic analysis identified three key themes from stage one interviews, central to participants’ experiences are identity, relationships and social networks. We found that time elapse from HCV cure did not alter participants’ views, meaning and values about treatment with DAAs and no new themes emerged from interview stages two and three.

#### Identity, relationships and social networks

Participants in our study described how their diagnosis of HCV negatively affected their health, well-being, and relationships. The stigma associated with HCV, due to its strong links with injecting drug use, contributed to a sense of having a “dirty” identity.

*“I mean it made me feel dirty with it…. the stigma with it*,* you know what I mean. Like your past and such*,* you take drugs and …” Participant 15*,* interview 1.*

This feeling of being “dirty” was compounded by fears of being perceived as contagious and a potential risk to loved ones. Children were felt to be particularly at risk from individuals diagnosed with HCV and there was a clear narrative of individuals feeling a responsibility to be careful to not pass it on.

*“Aye*,* I didnae want to go near my family unfortunately…….it was because of the kids n’ that aye” Participant 10*,* interview 1.*

The diagnosis often strained relationships and led to self-imposed isolation, which had a negative impact on mental health and weakened the support structures that were available for people to lean on in their recovery journey. After achieving cure, participants reported relief in shedding the “dirty” identity and no longer having to worry about transmitting HCV to loved ones, or even loved pets as described by one participant.

*“I’m glad it’s gone*,* as I say*,* I’m glad it’s gone. Ermm*,* I dare say if I had lived in a house with kids or something like that then I would have thought about it a lot more. Because obviously if you cut yourself or spot of blood lying about or something like that. Cause I live on my own*,* my dog. I used to*,* if I cut myself*,* you know how dogs like to lick your cut. I used to worry “god is my dog going to contract it.” That’s what I used to think*,* I used to worry about my dog contracting it.” Participant 3*,* interview 1*.

Once HCV cure was achieved participants reflected on the changing relationships within their family structure, often rebuilding relationships that had become distanced due to the threat of HCV’s contagious nature. They emphasised the importance of family support during treatment and expressed gratitude for this stability.

*“See what*,* it was good luck that way with my family as they have been really supportive. They are really good*,* and they’ve been the one constant thing that’s always been there for us. So was there for me the first time I caught it*,* and my mum was there for me when I finished.” Participant 12*,* interview 2*.

Treatment for HCV was seen as an opportunity for rebuilding relationships with family and friends, or for forming new relationships once the risk of transmission of infection was gone. Many participants linked HCV treatment with an improvement in their ability to form and sustain closer family relationships which supported their recovery journey.

*“Doing a lot more in my family. So*,* it’s like it’s definitely helped that that feeling” Participant 1*,* interview 1.*

Prior to HCV treatment we found that concerns about HCV transmission permeated everyday life. Misunderstandings about how HCV is transmitted heightened fears, with some participants avoiding everyday activities such as sharing cups and feeling the need to disclose their HCV status to their social network.

*“Errr*,* in some ways…. errr…I was more hesitant towards people and that…. And like the people*,* I actually telt people see how I had the hepatitis. I actually was open to people when I was gonna …. and that… and I used to have like my own sort of cup and that*,* I used to take a cup and that with me because they I mean like they share other cups ‘nd that maybe haven’t hepatitis and that eh…” Participant 16*,* interview 1.*

After participants were cured the threat of HCV remained for some with participants describing steps they are taking to ensure that they are not at risk of reinfection, limiting their social interactions and staying vigilant about situations that could pose a risk.

*“Oh definitely*,* just staying away from needles and don’t put myself in that predicament again. I can never really feel relaxed…. [interviewer: No*,* why? ] …Because you could never know when that situation could pop up. I’m always quite a bit cautious now when it comes to like using toothbrushes and razors and just anything drink (from)*,* you know*,* other people” Participant 12*,* interview 2.*

Social networks are recognised as a source of support, or as described by participant 14, a negative influence which may threaten hard earned recovery. Protecting their progress sometimes meant adopting a more isolated lifestyle.

*“Oh that….to be honest*,* I don’t have anybody that comes to my house because I got a new house and I don’t want to lose this house as it is probably in the best area of the city*,* so I stay clear so….no*,* no*,* no*,* no*,* no*,* I’m fine. Yeah*,* I’m fine.” Participant 14*,* interview 3*.

Some participants described their relationships with the drug treatment services. Whilst there was gratitude for the opportunities provide by HCV care to support them in their recovery there was frustration with the lack of support from drug treatment service. It was felt that their recovery efforts were not recognised and there was a lack of support to reduce their opiate substitution treatment.

*“Well I am still on my prescription of methadone…….but I have been asking and asking to come off it because I mean*,* I’m almost 50 years old and I don’t want to be on this anymore….but they are saying they can’t take me down because they haven’t got the help and support that I would need ….but they offered me more….but they wouldn’t put me down…and that is where I am with that….”Participant 14*,* interview 3*.

#### Building recovery capital

Participants reflected on their journey building recovery capital after achieving HCV cure. Many described feelings of loss associated with drug use and HCV, but following HCV treatment, they identified a range of activities and emotions that represented growth and a renewed sense of purpose. A key theme was the peace of mind that participants gained from being cured, with some participants feeling that HCV was firmly behind them.

*“God…I’ve never even thought about it for a long time now. You know what I mean? It’s not even been on my mind*,* so” Participant 3*,* interview 2.*

Other participants in the midst of treatment expressed optimism and hope for the future, voicing the expectation that HCV cure will lead to a shift in mindset and the ability to formulate plans for a life free from HCV.

*“Oh yeah*,* yeh*,* yeah*,* yeah*,* once it’s gone it will totally change my mindset [interviewer…. what do you mean by that? ] More positive about life because*,* I’m away to be 50 soon and I’ve not got too long left*,* so it’ll make a big difference to my life so….” Participant 14*,* interview 1*.

This sense of optimism was further solidified after they received the news that they had achieved sustained virological response (SVR) at 12 weeks post treatment. Participants reported engaging in activities that promoted their health and well-being, such as joining a gym or walking more frequently.

*“In general*,* I’ve joined the gym and I’ve put on a couple of stone n’ that so….” Participant 12*,* interview 2*.

*“Since I’ve been on treatment*,* I feel like I’m doing a lot more walks” Participant 1*,* interview 1.*

Moving to new housing or neighbourhoods was seen as a symbolic step toward leaving difficulties behind and embracing a fresh start.

*“…and we’ve moved house*,* moved in with my partner and we’ve got a ground floor flat in [names area] which is perfect.” Participant 15*,* interview 1.*

Physical health improvements were another key aspect of building recovery capital. Participants noted better appetite, weight gain, and an overall sense of vitality following treatment.

*“Errr*,* I feel errr like …… I am feeling fantastic aye*,* am feel really good feeling like I’m actually starting to put on the weight again I know I am feeling better*,* I can feel it in me. And it is strange because I didn’t even know I had it and then you realised you’ve got it and you can’t put on the weight but now that I am attending treatment*,* I am putting on the weight and I eat better” Participant 8*,* interview 1.*

Recovery held different meanings for our participants. For some, recovery extended to an expectation that they will achieve abstinence from drugs, with HCV cure serving as a turning point.

*“Well*,* I’m hoping that once I’m clear of that*,* that’s me off and totally away from the drugs.” Participant 1*,* interview 1*.

Others viewed HCV cure not as a direct catalyst for abstinence from drug use, but as a motivator to adopt harm reduction strategies, such as reducing injecting drug use or a shift to incorporate harm reduction measures into daily life which is part of their recovery journey continuum.

*“Ermm…………………I suppose I didnae really use needles as much now ken*,* ermm*,* before as well I used to share sometimes ken*,* ermm that completely stopped like aye*,* I’ve always made sure that I’ve got a fresh pack at the chemists whether I was going to use them or not*,* so it was always there sort of thing aye.” Participant 5*,* interview 1*.

Overall, participants’ reflections highlight the diverse ways in which they built or expected to build recovery capital through improved mental and physical health, renewed hope, strengthening relationships, and strategies for sustained recovery.

#### Reflecting on re-infection and the shift to DAAs

53% of people participating in our study were receiving DAAs as a result of re-infection with HCV. Participants often expressed a mix of gratitude and self-recriminations when discussing re-infection with hepatitis C. For those undergoing treatment for re-infection, feelings of undeservedness were common, as they attributed their condition to behavioural choices they regretted, rather than a lack of support in their recovery journey. This sense of vulnerability was particularly pronounced in discussions about the ease of treatment and the second chance provided by advances to DAAs.

*“I’m just glad to have managed to get treatment because really people have had it twice*,* really shouldn’t have*,* ken really*,* see really*,* well saying that its bad luck that you managed to go away and get it twice. It’s not like I went out really to catch it*,* do you know what I mean. I shouldn’t have been so stupid; I really shouldn’t have… I am just glad I have managed to get this treatment this time and it’s not so bad as the first treatment*,* because that was ughh. I don’t even know if I would do that again. I think I would have just left it.” Participant 1*,* interview 1*.

The shift from the older, gruelling interferon-based therapies to the more tolerable DAAs was a source of relief for participants, particularly those who had previous experienced the debilitating side effects of interferon. Many described their gratitude for the improved treatment options and acknowledged that without DAAs, they might have hesitated to seek treatment again.

*“I am just glad I have managed to get this treatment this time and it’s not so bad as the first treatment*,* because that was ughh. I don’t even know if I would do that again. I think I would have just left it. [Interviewer: That was interferon*,* wasn’t it? ] That was hard core that (laughs) that was the injection*,* that one that was really hard that one. I was constantly crying because I felt that ill every day. I couldn’t even eat brown sauce because it burnt all my mouth*,* everything and it was that nippy way. Curry*,* I couldn’t eat a curry. It was just horrible*,* scary horrible…. Yeah*,* I was really drained*,* and I went yellow*,* and I just didn’t…. I looked ill.” Participant 1*,* interview 1*.

Even participants who had no direct experience with interferon-based treatments expressed awareness of their severity, often contrasting it with the relative ease of DAAs.

*“Obviously*,* you know the old stuff was really bad. Interferon or something.” Participant 2*,* interview 1*.

The minimal side effects from DAAs were frequently highlighted as a key factor in participants’ willingness to re-engage with treatment after re-infection. This accessibility and tolerability were described as transformative, enabling participants to focus on recovery from drug use without the overwhelming burden of side effects.

*“Yeah*,* but the new stuff I’ve not really noticed. I haven’t had any side effects or anything…It’s pretty good” Participant 2*,* interview 1.*

In reflecting on re-infection and treatment, participants showed a deep appreciation for the advances in HCV care that allowed them to pursue treatment despite the emotional weight of their experiences with the disease.

## Discussion

Our thematic analysis identified three key themes: identity, relationships and social networks; building recovery capital; and reflecting on re-infection and the shift to DAAs.

Perceived contagiousness was a common thread across participants’ experiences, significantly impacting their sense of identity and their relationships. Many worried about transmitting HCV to others, particularly children and family members, which led to self-imposed distancing from loved ones. This behaviour often disrupted social roles and interactions, creating feelings of isolation [51, 52]. This self-imposed isolation, in turn, eroded the recovery capital they had to lean on by limiting their access to emotional and practical support available from their networks [25, 27, 52, 53].

Participants described the mental health toll of being diagnosed with HCV, particularly the stigma tied to their condition and the loss of connection to others. However, achieving HCV cure became a pivotal moment, allowing participants to rebuild relationships and regain the support of social networks. Family bonds, in particular, were strengthened once the risk of transmission was eliminated, highlighting the transformative effect of HCV treatment on identity and interpersonal dynamics. These findings align with previous qualitative studies emphasising the restorative potential of HCV cure for individual’s social roles and relationships [20, 54, 55].

HCV cure was suggested to facilitate personal growth, as participants described gains in identity, aspirations, and social connectedness. Many expressed renewed optimism for the future, linking HCV cure to expectations for future plans and employment opportunities. Participants with children envisioned a more active role within family life. Those who had realised their HCV cure found they were now able to undertake activities that improved their health and well-being. Moving to a new house or neighbourhood was also seen as a symbolic step towards leaving past difficulties behind and embracing a fresh start.

These positive changes enhanced participants’ recovery capital, providing resources to support a recovery journey [25, 27]. Importantly, these gains were not contingent on complete abstinence from substance use, as demonstrated by the WHO ASSIST measure describing participant demographics. This perspective reinforces the idea that recovery is a process that encompasses other markers of progress, such as improved physical health, mental well-being, and the strengthening of social bonds [24].

The inclusion of participants who were receiving DAAs due to re-infection allows us to consider the experiences of this population. There is a profound sense of gratitude for the opportunities afforded to people through testing and treatment and the ripple effect to their wider life. This gratitude was particularly pronounced among those receiving DAAs for re-infection, as they often described feeling undeserving of care due to perceiving HCV as a consequence of their own actions and their failure to make the required behavioural changes previously. These feelings echo findings from other research, which highlight how people with HCV may internalise stigma, view their diagnosis as a personal failing and feel undeserving of the investment of treatment [56–61].

The shift to DAAs was a source of relief and gratitude for participants. Even those without firsthand experience of interferon-based treatments were acutely aware of its treatment burden and significant side-effect profile [6, 7, 19]. The reputation of the interferon-era has been passed on by others as a way of making sense of the social context of HCV treatment [62]. The minimal side effects of DAAs were credited as a key factor in engaging with treatment in line with findings from other research [63, 64]. This accessibility enabled participants to focus on their recovery journey without the physical and emotional toll of interferon, underscoring the importance of DAAs in expanding treatment access and reducing barriers. The limited longitudinal follow up of individuals receiving treatment for re-infection prevents us from determining whether this round of DAA treatment will be sufficient to alter risk behaviours that thus far have been deeply ingrained.

Our results describe an interplay of identity, relationships and recovery capital that deliver non-clinical outcomes for people treated with DAAs. Our key themes reflect the conceptual findings of other literature that describes the anticipated and actualised outcomes of DAA treatment [20, 54]. The stigma and isolation associated with HCV shaped participants’ identities, while DAA treatment supported them to rebuild relationships and reclaim social roles. Recovery capital was enhanced through strengthened family bonds, improved physical and mental health and well-being, and renewed aspirations for the future which provides a foundation for recovery. Gratitude for the transformative potential of DAA treatment permeated participants’ narratives, offering a hopeful perspective on their recovery journeys. The newfound opportunities to build support for their recovery and the appreciation for effective accessible DAA treatment underscored the multifaceted impact of curing HCV on identity, relationships, and recovery.

Work by Kagan et al. [35] however cautions that “the hepatitis C label is not always cleared” with a lingering sense of shame and embarrassment. These descriptions permeated our findings, where participants reflected that they felt gratitude for treatment even if they judged themselves unworthy recipients at the time, particularly if receiving treatment for reinfection. Our study also resonates with “living with untidy endings”, identified in the same study, where participants identified unmet needs in terms of support available to cope with reintegration into normal life [35]. Participants in our study expressed frustrations with drug treatment service support, and whilst many were able to voice aspirations for change, they do not always match what happens in reality. Social isolation in the wider community was still apparent for those who had finished treatment in our study. Many participants described a reduction in social connection to people who actively use drugs, demonstrating the early stages of change in the SIMOR [28–30]. This was also captured through the social identity mapping exercise where participants described a general shift away from people who actively use drugs. There was little evidence of participants’ increasing social connections beyond immediate family members towards others in recovery, or unconnected to drug use, described as a stable recovery position by SIMOR [28].

Our study finds that there are unmet needs in terms of the post-cure pillar in the HCV cascade of care that links people into their communities to deliver social redemption and reconnection and support recovery journeys [34]. To maximise the benefits of HCV cure, healthcare providers should consider what opportunities they can provide to link into other social networks as part of the cascade of care, for example through social prescribing and link workers [65]. Social prescribing initiatives connect people to community-based support, activities and services that meet the needs of the individual and encourage important community links [65].

Our findings contribute to formulating and building a theory of the influence treatment with DAAs has in the wider recovery journey. This is increasingly important as uptake of DAA treatment remains stubbornly behind targets in some areas [15–17, 66]. Building understanding of how DAA therapy can deliver outcomes that are important to PWID, such as recovery, social redemption and a new identity may provide compelling evidence to support a renewed effort to each the WHO 2030 HCV elimination goal [20, 54, 55, 61].

### Limitations

There are several limitations to our study. The study was conducted during the Covid-19 pandemic and social distancing requirements may have impacted on the researcher’s ability to form a trusting relationship with participants. A large proportion of participants were lost to follow up. As NHS Tayside moved towards elimination a possible explanation for lack of engagement may have been that the pool of potential participants consisted of individuals that thus far had been difficult to reach, this may have impacted on study recruitment and retention. More than half of participants were receiving DAAs due to reinfection, suggesting that they may have faced greater challenges in making life changes, thereby complicating longitudinal follow-up efforts.

## Conclusion

Our study provides insights into the views, meaning and value people who use drugs place on DAA treatment and the influence on recovery journeys. The themes identified in our study describe the initial stages of a recovery journey, an improvement in health and wellbeing as a result of HCV cure through the social identity model of recovery. Individuals reduced the number of active users within their social network and reconnected with immediate family members, building support for their recovery journey. However, we found that individuals remain relatively socially isolated in the context of the wider community at this stage. Further longitudinal research is required to ascertain the impact of HCV cure on the maintenance of recovery. HCV services should make links with wider community resources to support PWID to establish wider community contacts that may aid this process. HCV services should also strengthen links with drug treatment services to support people achieve their aspirations from treatment after HCV cure is achieved in the post-cure pillar in the cascade of care.

## Electronic supplementary material

Below is the link to the electronic supplementary material.

Supplementary Material 1

Supplementary Material 2

## Data Availability

The qualitative data that support the findings of this study are not openly available due to reasons of sensitivity and are available from the corresponding author upon reasonable request.

## Abbreviations

HCV: Hepatitis C Virus
SIMOR: Social Identity Model of Recovery
DAA: Direct Acting Antiviral
WHO: World Health Organization
PWID: People Who Inject Drugs
WHO ASSIST: World Health Organization Alcohol, Smoking and Substance Involvement Screening Test
SWEMWBS: Short Warwick–Edinburgh Mental Wellbeing Scale
SIM: AIRing–Social Identity Map–Ascertaining Identity Resources
SPQR: Standards for Reporting Qualitative Research
