# Supplementary material for: Future destinations: how people cured of hepatitis C using direct acting antiviral drugs progress in a new HCV-free world. A thematic analysis
Source: Harm Reduct J. 2025 Jan 16;22:10. doi: 10.1186/s12954-024-01142-3 (PMC11737260; doi:10.1186/s12954-024-01142-3)
Supplement: Supplementary file 1 — Supplementary Material 1 [file 12954_2024_1142_MOESM1_ESM.docx]

## **Supplementary Material Table 1 Semi-structured Interview Tool**
